# The Effect of Being Aerobically Active vs. Inactive on Cutaneous Vascular Conductance during Local Heat Stress in an Older Population

**DOI:** 10.3389/fphys.2017.00859

**Published:** 2017-10-31

**Authors:** Ulrike H. Mitchell, Samantha Burton, Christopher Gordon, Gary W. Mack

**Affiliations:** Department of Exercise Sciences, Brigham Young University, Provo, UT, United States

**Keywords:** microdialysis, skin blood flow, cutaneous vascular conductance, nitric oxide, aerobic training, elderly

## Abstract

**Objective:** To test the hypothesis that long- term aerobically trained elderly individuals have a greater amount of bioavailable nitric oxide (NO) and have a larger cutaneous vasodilation during local heat stress compared to their inactive elderly counterparts.

**Methods:** Eight aerobically trained and 8 inactive older men (>60 years old) participated in this study. NO bioavailability in blood and intradermal dialysate were measured with an ozone based chemiluminescence NO analyzer. Cutaneous vasodilator response to local heating was obtained using laser Doppler velocimetry.

**Results:** Whole blood NO were similar in older- trained and inactive subjects (0.75 ± 0.56 and 0.38 ± 0.32 μM, respectively; Mann–Whitney, *p* = 0.153), as was intradermal dialysate NO before (7.82 ± 6.32 and 4.18 ± 1.89 μM, respectively) and after local heating (7.16 ± 6.27 and 5.88 ± 3.97 μM, respectively, *p* = 0.354). The cutaneous vasodilator response of the older- inactive group was smaller than the older- trained group [Group-Time interaction, *F*_(24, 264)_ = 12.0, *p* < 0.0001]. When compared to a young group the peak vasodilator response of the older- trained subjects was similar. However, the time to initial dilation was 3.1 and 2.2 times longer (*p* < 0.05) in older- inactive and older- trained subjects, respectively, compared to young subjects.

**Conclusions:** Our data support the hypothesis that the age-related reductions in cutaneous vasodilation can possibly be restored by maintaining an aerobic training regimen (at least 3 years). However, some residual effects of aging remain, specifically a delayed cutaneous vasodilator response to local heating is still present in active older adults. We found no evidence for an increase in systemic or local NO-bioavailability with an extended commitment to aerobic fitness.

## Introduction

Nitric oxide (NO) is a vasoactive molecule that can cause an increase in blood flow and oxygen delivery to tissue. Increased bioavailability of NO through exercise training (Clarkson et al., 1999; Kingwell, 2000; Taddei et al., 2000; Franzoni et al., 2004; Maeda et al., 2004; Zago et al., 2010b) improves vascular function (Kingwell, 2000) and blood pressure regulation (Zago et al., 2010a,b), which can lead to a range of health benefits, especially in the elderly. A reduction in NO bioavailability can reduce oxygen delivery and contribute to the development of hypertension (Calver et al., 1992), coronary artery disease (Lefroy et al., 1993), and/or congestive heart failure (Moncada et al., 1991). Reduced NO bioavailability is related, in part, to a decline in the activity of vascular endothelial nitric oxide synthase (eNOS) (Taddei et al., 2001). This is typically observed in adults over the age of 60 years (Armstrong and Kenney, 1993; Taddei et al., 2001; Pierzga et al., 2003) and can be reversed by pharmacologically improving NOS activity (Holowatz et al., 2007).

Age-related declines in NO bioavailability are attenuated with 3 months of mild aerobic exercise training in older adults >60 years old (Maeda et al., 2004) and eNOS activity is increased in pre-hypertensive older subjects after 6 months of exercising (Zago et al., 2010a). Evidence supports the hypothesis that the augmented eNOS activity is a function of increased vascular shear forces during exercise (Ballermann et al., 1998). Increased eNOS activity, and thereby NO bioavailability in the cutaneous circulation, is evaluated by the cutaneous vasodilator response to local heating. The peak increase in skin blood flow (SkBF) during 30–40 min of local heating (~42°C) is primarily mediated (>90%) by NO (Minson et al., 2001). The plateau in SkBF during local skin heating is lower in older adults and reflects a reduction in eNOS-mediated vasodilation (Kenney, 1988; Armstrong and Kenney, 1993; Kenney and Ho, 1995).

NO plays a major role in the cutaneous vasodilator response to local skin heating. This response is decreased in the elderly (Kenney, 1988; Armstrong and Kenney, 1993; Kenney and Ho, 1995), but can be partially restored by short term aerobic training. It is therefore presumed that elderly individuals, who maintained an active, aerobically trained lifestyle, demonstrate an increase in NO-bioavailability and a greater increase in SkBF during local heating compared to less active elderly individuals. Franzoni et al. (2004) assessed plasma NOx concentrations in young and old athletes, using a colorimetric Griess assay, and compared the results to matched sedentary controls. We wanted to build upon that study by measuring skin interstitial nitrite levels in addition to plasma NOx and by using chemiluminescence to detect NO. The purpose of our study was therefore to quantify NO bioavailability, via blood and skin interstitial nitrite levels (Beckman and Koppenol, 1996) in a population of long-term aerobically trained elderly men as compared to a group of age-matched, inactive elderly individuals. In addition, we measured the cutaneous vasodilator response to local skin heating as a bioassay for cutaneous dilation. We tested the hypothesis that active, trained elderly individuals have a greater amount of bioavailable NO and have a larger cutaneous vasodilation during local heat stress compared to their inactive elderly counterparts.

## Materials and methods

### Subjects

We recruited men over the age of 60 years. Power analysis indicated that we should be able to detect a 15% difference in the skin blood flow response to local heating using 8 subjects per group. Inclusion criteria for all subjects consisted of being generally healthy, normotensive or pre-hypertensive, nonsmokers, non-diabetic, and not taking any medications that could influence cutaneous blood flow responses to local heating.

Eligible subjects were initially screened via phone interview to assess if they fit into either the “aerobically trained” or “inactive control” group. We asked detailed questions about their physical activity habits—type of exercise, frequency, and how many years they have consistently participated. To be considered for the aerobically trained group the subjects had to exercise at least 3 times per week over at least the previous 3 years. To be considered for the inactive control group, the subjects had to report no pattern or significant amount of physical activity in the last 3 years. Twenty-nine volunteers fell into one of these groups, and they were invited to continue with the study and to undergo further screening.

This study was carried out in accordance with the recommendations of federal ethics guidelines, Code of Federal Regulations, TITLE 45, PUBLIC WELFARE, DEPARTMENT OF HEALTH AND HUMAN SERVICES, PART 46, PROTECTION OF HUMAN SUBJECTS with written informed consent from all subjects. All subjects gave written informed consent in accordance with the Declaration of Helsinki. The protocol was approved by Institutional Review Board at the authors' university, and each subject gave written informed consent before participation. Subjects who volunteered to participate in this study were asked to fill out a Rapid Assessment of Physical Activity questionnaire (Topolski et al., 2006). This questionnaire was used to determine a basic, self-reported physical activity level. This assessment focused primarily on current activity level. If their responses placed them in the inactive category (score of 1 or 2) or the trained category (score 6 or 7) they met the first criterion for the study and were referred to the physician for further consideration. Eleven subjects scored a 3, 4, or 5 on the questionnaire and were excluded from the study.

A physician reviewed the health history and medication usage of each person. If they had a significant health condition that may have affected the results of this study (heart condition, disease, diabetes, or major surgical history) or were using medication that alters blood flow, they were excluded from the study. Blood pressure, heart rate, height, and weight were measured as population descriptors. All remaining subjects were cleared by the physician to proceed with the sub-maximal treadmill exercise test. The purpose of this test was to estimate $\dot{V}O_{2}$max and to verify that the subjects' perceived level of physical activity matched the appropriate level of aerobic fitness level. Heart rate was measured during treadmill exercise using an electronic heart rate monitor (Polar Heart Rate Monitor, Polar Electro Inc., Lake Success, NY) and maximal heart rate was estimated using the equation (Robergs and Landwehr, 2002): HR_max_ = 205.8–0.685^*^(age).

The submaximal exercise test began by walking for 4 min at a 0% grade at a self-selected comfortable speed (usually between 2 and 4.5 mph). Next, the grade of the treadmill was increased to 5% while the speed was maintained. This pace and grade was maintained for 4–5 min with the heart rate of the patient measured at the end of minutes 3, 4, and 5. The average HR between minutes 3 and 5 was used to estimate $\dot{V}O_{2}$max using the following equation (Vehrs et al., 2007): $\dot{V}O_{2}$max (ml O_2_•kg^−1^•min^−1^) = 58.687 + (7.520 × Gender; 0 = woman and 1 = man) + (4.334 × mph) − (0.211 × kg) − (0.148 × HR) − (0.107 × Age).

All subjects reported to the laboratory for the second visit after having fasted for 8 h. Using a glucometer fasting glucose levels were measured. Two subjects' fasting glucose levels were higher than 126 mmol • Liter^−1^ and were excluded from the study (Centers for Disease Control Prevention, 2011). A 3 ml blood sample from an antebrachial vein was then drawn into a Li-heparin vacutainer for measurement of NO concentration. The blood sample was immediately combined with a nitrite preservation solution (0.8 M ferricyanide, 0.1 M N-ethylmaleimide, and 500 uL IGEPAL®CA-630) in a 4:1 (v/v) ratio. The solution was gently mixed by inversion and stored at −80°C until analysis.

The subjects were divided into two groups: aerobically trained [*n* = 8, age = 66 ± 5 years, body mass index (BMI) = 25.7 ± 1.7 kg•m^−2^, estimated $\dot{V}O_{2}$max of 39.1 ± 1.2 ml • min^−1^ • kg^−1^] and inactive controls (*n* = 8, age = 66 ± 9 years, BMI = 30.1 ± 4.6 7 kg•m^−2^, estimated $\dot{V}O_{2}$max of 29.0 ± 2.7 ml • min^−1^ • kg^−1^).

### NO analysis

Blood and dialysate nitrite concentrations, our proxy for NO bioavailability were measured, using an ozone based chemiluminescence NO analyzer (Sievers Instruments, Boulder, CO, USA). Before analysis, the NO analyzer was calibrated according to the manufacturer protocols using 8 levels of sodium nitrite (0, 0.01, 0.05, 0.1, 0.5, 1.0, 10.0, and 50 μM). The glass purge vessel contained a tri-iodide solution that consisted of 50 mg potassium iodide, 2,000 μl of ultrapure water, and 5 ml of glacial acetic acid. Each frozen sample was rapidly thawed in a 37° C water bath, deproteinated with 100% methanol (1:1 v/v) and then centrifuged at 15,000 rpm for 3 min. The supernatant was removed and placed in a clean nitrite free Eppendorf tube. The Eppendorf tube was then centrifuged again and the supernatant transferred to a second nitrite free tube for analysis. NO analyses were performed in triplicate using 100 μl of the supernatant of the deproteinized blood sample and 10 μl of the supernatant of the deproteinized dialysate. The area under the NO-time curve was used to determine nitrite concentration based upon our 8-point calibration curve. The average coefficient of variation triplicate measures of the standards and samples were 11 and 23%, respectively.

### Cutaneous vasodilator response to local heating

Two linear intradermal microdialysis probes (2.5 cm hollow fiber with molecular weight cut-off of 18 kDa) were placed in the dermis of the skin of the dorsal aspect of the non-dominant forearm at <1 cm apart. The single use microdialysis probes were assembled in our laboratory, following guidelines established in previous research (Coglianese et al., 2011) and gas sterilized before use. Placement of the microdialysis fibers was performed by first inserting a 27 g 3.50 inch needle just underneath the skin (≈1–2 mm depth) with entry and exit sites ~3 cm apart. The microdialysis probes were threaded through the needle. Once the probes were threaded, the needle was carefully removed, leaving the probe in place. A 3 × 3 cm Peltier-controlled thermomodule was placed directly over the two microdialysis probes with a laser Doppler flow probe (model: VP7a, Moore Instruments, Wilmington, DE) placed in a central access point in the Peltier. The Peltier was secured to the forearm using a double stick disc. The laser Doppler output and thermomodule temperature were monitored using a Powerlab system (ADInstruments Inc., Colorado Springs, CO). The microdialysis probes were flushed with sterile saline (5.0 μl/min) for about 90 min with an infusion pump (model VPF; Harvard Apparatus, Holliston, MA) while local skin temperature under the thermomodule was held at 32°C. An automated noninvasive brachial blood pressure monitor (Tango+, SunTech Medical Instruments, NC, USA) was placed on the dominant arm, opposite of the microdialysis site. The arm was placed in a relaxed, pronated position at heart level. Blood pressure was recorded every 5 min throughout the local skin heating protocol. After 90 min of flushing with saline the perfusion rate of the microdialysis probes was reduced to 1 μl/min and the dialysate from the two microdialysis probes were collected into a 1.5 ml amber Eppendorf tubes containing the nitrite preservation solution in a 4:1 ratio (v/v) over a 30 min period. The thermomodule temperature was then increased to 42°C at a rate of 0.1°C per second and was held at 42°C for 40 min. A second dialysate sample was collected during this 40-min heating period while skin blood flow was continuously recorded using laser Doppler flowmetry. The dialysate samples were gently inverted and frozen at −80°C until analysis.

After 40 min of local heating at 42°C the microdialysis probes were perfused with 28 mM sodium nitroprusside (SNP) at 5 μl/min until a second plateau in SkBF was reached, while maintaining the skin temperature at 42°C. Twenty-eight millimolars of SNP at a skin temperature of 42°C is sufficient to elicit a maximum cutaneous vasodilation (Minson et al., 2002). After a plateau was reached perfusion of SNP was stopped and the blood pressure cuff placed on the dominant arm was inflated to >200 mmHg to verify zero SkBF. Vasomotor activity in the skin was expressed as cutaneous vascular conductance (CVC) by dividing SkBF measurements (volts) by the average mean arterial blood pressure during the local heating protocol. These data were normalized to maximal CVC obtained with 28 mM SNP and then expressed as percent of CVC_max_ (%CVC_max_).

### Data analysis

After data collection one inactive control subject disclosed taking medication that might have altered the skin blood flow response to heating. As such, his data were removed from analysis. For comparison a young control group (24 ± 2 years, 170.8 ± 2.9 cm, 69.4 ± 3.6 kg) was included in this study for comparison to the older subjects, these data have been previously reported (Mack et al., 2016). A two-way ANOVA was performed on the %CVC_max_ to compare the difference between young, older -trained and older -inactive individuals. The raw data for both the blood and the dialysate nitrite concentrations were not normally distributed. Whole blood nitrite concentrations were analyzed using an unpaired Mann–Whitney test. The pre- and post-heating dialysate samples were normalized using a log transform and then compared using a two-way ANOVA. A two-way ANOVA was performed on the %CVC_max_ to compare the difference between young, older -trained and older -inactive individuals. The level of significance was set at *p* < 0.05.

## Results

### Subjects

The older subject characteristics are presented in Table 1. The average age, BMI, diastolic blood pressure, and fasting blood glucose were similar in both trained and inactive older groups. However, the older-trained subjects had a higher estimated $\dot{V}O_{2}$max and a lower resting systolic blood pressure than the subjects in the inactive group (*p* < 0.05).

**Table 1:** Older subject characteristics.

**Group**	**Age years**	**BMI kg•m^−2^**	**SBP mmHg**	**DPB mmHg**	**Glucose mmol•L^−1^**	**Estimate $\dot{V}O_{2}\text{max}$ ml O_2_•kg^−1^•min^−1^**
Trained (*n* = 8)	66 ± 5	25.7 ± 1.7	113 ± 7^*^	69 ± 5	98 ± 9	39.1 ± 1.2^*^
Inactive (*n* = 7)	66 ± 9	30.1 ± 4.6	124 ± 13	72 ± 8	116 ± 9	29.0 ± 2.7

Values represent mean ± 1 SD for each group. BMI, body mass index; SBP, systolic blood pressure; DBP, Diastolic blood pressure; $\dot{V}O_{\text{2}}max$, maximal oxygen consumption.

* *p < 0.05 different from untrained*.

### NO concentration

Whole blood NO concentration was similar in older- trained and inactive subjects averaging 0.75 ± 0.56 and 0.38 ± 0.32 μM, respectively (Mann–Whitney, *p* = 0.153). The nitrite levels in dialysate samples collected at a skin temperature of 32°C were similar in both older groups, trained and inactive, and averaged 7.82 ± 6.32 and 4.18 ± 1.89 μM, respectively. During 40 min of local skin heating to 42°C, the dialysate nitrite level did not increase in either trained or inactive older subjects averaging 7.16 ± 6.27 and 5.88 ± 3.97 μM, respectively [*F*_(1, 13)_ = 0.923, *p* = 0.354]. When the data of the two groups were pooled a Wilcoxon matched-pairs signed rank test did not uncover any increase in dialysate nitrite concentration with local skin heating (*p* = 0.99).

### Cutaneous vasodilator response to local heating

**Figure 1:**
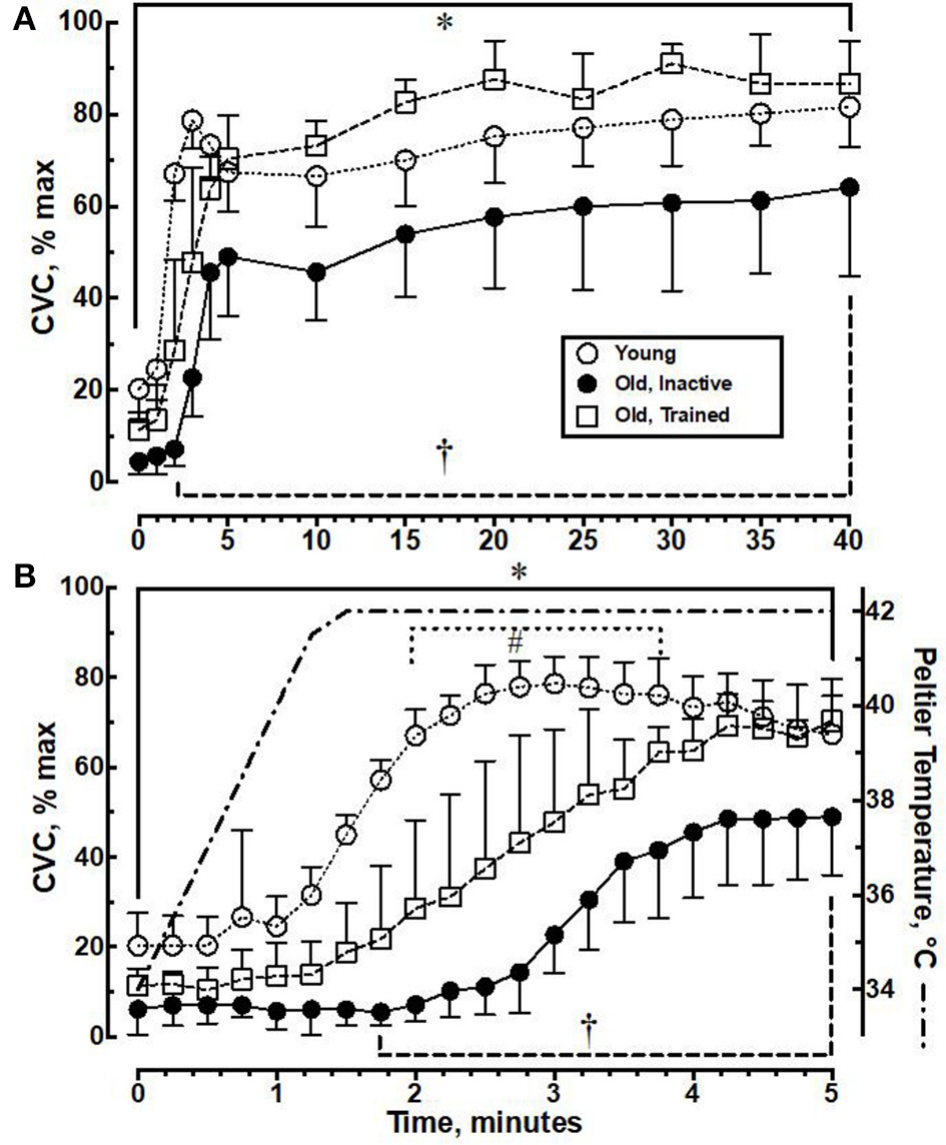
**(A)** Cutaneous vascular conductance (CVC) changes, expressed as a percentage of maximal CVC (CVC % max), during 40 min of local skin heating to 42°C. **(B)** The first 5 min of CVC data replotted to illustrate the initial time course of change following the onset of local heating and the corresponding Peltier temperature. Values represent Mean ± 1 SD for 10 young, 8 old- trained, and 8 old- inactive subjects. ^*^p < 0.05 young different from older- inactive, ^†^*p* < 0.05 older- trained different from older- inactive. ^#^*p* < 0.05 young different from older- trained.

Figure 1 illustrates the CVC response to local skin heating to 42°C for 40 min in young, older trained, and older inactive subjects. The general characteristics of the cutaneous vasomotor response to local skin heating are given in Table 2 as previous described by Minson et al. (2001). The primary parameter of interest is the plateau portion which reflects primarily NO-mediated dilation. The older- inactive group's plateau was 18–24% lower than the young or older-trained group [*F*_(2, 22)_ = 18.4, *p* < 0.0001]. In general, the vasodilator response of the older trained subjects was similar to that of the young control group (Figure 1A). The cutaneous vasodilator response of the older- inactive group was lower than both the young control and older- trained group [Group-Time interaction, *F*_(24, 264)_ = 12.0, *p* < 0.0001]. Detailed inspection of the data revealed that during the first 5 min of local heating there were also some important differences between the young and older- trained groups (Figure 1B). Specifically, a significant interaction between time and treatment group [*F*_(40, 460)_ = 11.0, *p* < 0.0001] identified a lag in the rise in CVC in the older- trained group compared to the young control group with an even greater lag in the older- inactive group. Table 3 provides the response time analysis of the CVC response to the first 5 min of local skin heating. The time it took to initial an increase in CVC during local skin heating (threshold) was 2.2 times longer in older- trained subjects compared to young (*p* < 0.05). Older- inactive subjects required 3.1 time more time to initial dilation (*p* < 0.05). Overall the response time, the time from the start of heating to when CVC reached 63.25% of the plateau was 1.6–1.8 times longer in the older- trained and older- inactive groups, respectively (*p* < 0.05). The span in %CVCmax (the difference between the baseline and peak CVC during the first 5 min) was similar in young and older- trained groups. However, the span was significantly reduced in the older- inactive group (*p* < 0.05).

**Table 2:** Cutaneous vasomotor response to 40 min of local skin heating to 42°C.

**Group**	**Baseline, %CVCmax**	**Initial peak, %CVCmax**	**Nadir, %CVCmax**	**Plateau, %CVCmax**
Young (*n* = 10)	20.3 ± 6.9	80.6 ± 5.6	57.7 ± 9.4	80.4 ± 8.0
Older-trained (*n* = 8)	11.6 ± 2.5^*^	77.0 ± 7.3^†^	43.0 ± 16.4^*^^†^	86.3 ± 8.6^†^
Older-inactive (*n* = 7)	5.8 ± 3.6^*^	53.0 ± 13.3^*^	22.2 ± 7.8^*^	62.7 ± 17.6^*^

Values represent Mean ± 1 SD. CVC, cutaneous vascular conductance expressed as a percent of maximal (%CVCmax);

* p < 0.05 different from young.

† *p < 0.05 different from old- inactive. The data for the young group were previously reported (Mack et al., 2016)*.

**Table 3:** Response characteristics of the cutaneous vasodilation during the first 5 min of local skin heating to 42°C.

**Group**	**Threshold, min**	**Time constant, min**	**Span, %CVCmax**	**Response time, min**
Young (*n* = 10)	0.7 ± 0.2	1.1 ± 0.3	60.2 ± 10.1	1.8 ± 0.2
Older-trained (*n* = 8)	1.6 ± 0.9^*^	1.3 ± 0.4	64.3 ± 8.5^†^	2.9 ± 0.8^*^
Older-inactive (*n* = 7)	2.2 ± 0.4^*^	1.2 ± 0.3	45.6 ± 13.9^*^	3.4 ± 0.2^*^

Values represent Mean ± 1 SD. CVC, cutaneous vascular conductance expressed as a percent of maximal (%CVCmax); min, minute, Time Constant, the time required to reach 63.2% of the Span, Span, difference in CVC from baseline to 5 min, Response time, time from start of heating till CVC reaches 63.2% of the Span and Response time, equals the time constant + threshold.

* p < 0.05 different from young.

† *p < 0.05 different from older- inactive. The data for the young group were previously reported (Mack et al., 2016)*.

## Discussion

The significant and novel observation of this study was that older individuals, who maintained a 3-year or greater history of aerobic exercise, were able to preserve the peak cutaneous vasodilator response to local skin heating (Figure 1A). However, the older- trained group still demonstrated some important differences in the cutaneous vasodilator response during the first 5 min of local skin heating (Figure 1B). These data indicate that a history of aerobic training does not wholly prevent deterioration in the cutaneous vascular responses to local heating. These data support the hypothesis that older individuals, who maintain a regimen of aerobic exercise, can preserve the cutaneous vasodilator response to local heating. Despite our ability to demonstrate a physiologically important maintenance of cutaneous dilation during local skin heating, we were unable to identify any systemic or local difference in nitrite levels (blood or intradermal dialysate) that may have indicated an increase in NO bioavailability in the older- trained group. The reduction in cutaneous vasodilator response to thermal stress (local or whole body) with aging has been confirmed by several studies (Kenney, 1988; Minson et al., 2002; Holowatz et al., 2003, 2007). Specifically, several studies have documented a reduced cutaneous vasodilator response to local skin heating in older individuals (Minson et al., 2002; Holowatz et al., 2003; Lang et al., 2009). Minson et al. (2002) examined the NO-mediated cutaneous vasodilator response to local skin heating in groups of young and old individuals. In the older group (5 men and 5 women, ranging in age from 69 to 84 years) the plateau CVC during 40 min of local heating averaged 82 ± 5 %CVCmax which was ≈12% lower than the young group (93 ± 2 %CVCmax). We noted a similar reduction in %CVC max in our older- inactive group compared to a group of young adults. However, this reduced cutaneous dilation in response to local heating was eliminated in the older- trained men. While the plateau CVC during local skin heating was preserved in the older- trained subjects the time course for the cutaneous vasodilator response was impacted by aging. First, baseline CVC at a skin temperature of 32°C was lower in both older groups compared to the young subjects. The initial peak CVC response to the onset of heating (Table 2), which occurs during the first 5 min of local heating, was lower in the older- inactive compared to either the older- trained or the young group. We evaluated this initial vasodilator response (Figure 1B and Table 3). The onset of cutaneous vasodilation in the young group occurred when the Peltier temperature reached slightly over 38°C. In contrast, in the older- inactive group the threshold for cutaneous vasodilation did not occur until 42 s following the Peltier temperature had reached 42°C. The older- trained group initiated cutaneous dilation about when the Peltier heater reached 42°C. The delay in cutaneous dilation is most likely a result of an impaired sensory detection of changes in skin temperature (Stevens and Choo, 1998) and/or attenuated activation of local transient receptor potential receptors (TRP receptors) in the skin (Wong and Fieger, 2010).

Studies have shown improved vasodilator responses in elderly subjects following several weeks of aerobic training (DeSouza et al., 2000; Galetta et al., 2006). Our data are consistent with these findings when we compare the older- trained and older- inactive groups. Our data go further by indicating that long term participation in regular aerobic training, (3 years or longer) seems to prevent most of the expected decline in cutaneous vascular response to local heating.

We had hypothesized that the improved cutaneous vasodilator response to local heating is associated with an increase in NO bioavailability. This was, in part, based on the findings by Franzoni et al. (2004) who found a significantly higher NOx plasma concentration in older trained subjects when compared to matched sedentary controls. Maeda et al. (2004) had similar results after they placed 7 sedentary older women (59–69 years old) on a 3-month aerobic exercise program. The authors reported a significant increase in [NOx]_blood_ compared to baseline and compared to a control group (*p* < 0.01 for both). The two aforementioned studies used chemical analysis (Griess reaction) to obtain their data, while we used an ozone-based chemiluminescence detector. Our data on blood and intradermal dialysate nitrite concentrations in the older- trained and inactive groups did not support our hypothesis. Nevertheless, our data are consistent with the results from Zago et al. (2010b) who also did not find a change in [NOx]_blood_ in 16 subjects (≈59 years old, male and female) after they participated in a 6-month aerobic exercise program.

Using an amperometric NO sensor Clough et al. (Clough et al., 1998; Clough, 1999) reported basal concentrations of NO in intradermal dialysate of 0.63 ± 0.09 and 0.49 ± 0.06 μM. The range of intradermal NO reported was 0.73–4.3 μM. Kellogg et al. (2003), using similar methods, reported basal values of diffusible NO in cutaneous interstitial tissue of 0.54 ± 0.11 μM. In both of these studies, the amount of NO present in the tissue increased in response to heat and/or pharmacological agents known to activate the NOS pathway. In contrast, using chemiluminescence determination of [NOx] in intradermal dialysis, Crandall and MacLean (2001) reported basal values of [NOx]_dialysate_ of 7.6 ± 0.5 μM that did not increase with whole body heating but did increase when stimulated with acetylcholine. In the current study the baseline levels of [NOx]_dialysate_ ranged from 1.16 to 19.52 μM with a pooled average of 6.12 μM for both older groups prior to heating which was similar to that reported by Crandall and MacLean (2001). However, [NOx]_dialysate_ did not increase with local skin heating. The inability to detect an increase in [NOx]_dialysate_ during local heating may be a reflection of a high basal levels of [NOx]_dialysate_, a short half-life of NO, or the scavenging of NO by several biological substrates (beside conversion to nitrite).

The physiological significance of our findings is that age-related declines in cutaneous vasodilation in response to local heating are eliminated in individuals who participate in chronic endurance exercise. While cutaneous vasodilation is the result of synergistic activation of multiple mechanisms, the NO-dependent portion accounts for ≈60% of the response in older subjects (Holowatz et al., 2003). We therefore, believe that improved NOS activity is at least partially responsible for the improved cutaneous vascular response with chronic endurance exercise. However, we were unable to confirm an increased bioavailability of NOx. Based on our findings chronic endurance exercise in aging subjects should mitigate microvascular dysfunction associated with aging.

### Limitations

Using only men in this study could be perceived as a limitation of this study, especially in regards to generalizability. We decided to limit our study to men to avoid the confounding influence of sex on NO bioavailability. It is known that menopausal status (premenopausal versus postmenopausal) and use of hormone replacement therapy can impact NO bioavailability (Tehrani et al., 2015). We did not control for intake of dietary sources of NOx over the days prior to testing; this could have skewed our results, as bioavailability of NOx can be affected by diet.

## Conclusion

Our data support the hypothesis that the age-mediated reductions in cutaneous vasodilation seen in inactive older adults might be preventable by maintaining a long-term (at least 3 years) aerobically training regimen. However, despite the ability to fully dilate the cutaneous vasculature during local heating some residual effects of aging remain, specifically we noted that the response time to local heating is slower in both inactive and aerobically-trained older adults. Despite the improvement in cutaneous dilation in the aerobically-trained older adults we did not detect any significant differences in systemic or local NO-bioavailability. This latter result may reflect a measurement problem associated with the extremely short half-life of locally released NO. Overall, we can conclude that an extended commitment to aerobic fitness (at least 3 years) is associated with an attenuation of the typical age-related dysfunction of the cutaneous vasodilation system.

## Author contributions

UM, GM, and SB: Conception and Design, Collection and data assembly, Interpretation of results, Manuscript writing and Approval for submission; CG: Collection and data assembly, Interpretation of results, Manuscript writing and Approval for submission; UM: Financial support.

### Conflict of interest statement

The authors declare that the research was conducted in the absence of any commercial or financial relationships that could be construed as a potential conflict of interest.
